# Breastfeeding assessment tools for at-risk and malnourished infants aged under 6 months old: a systematic review

**DOI:** 10.12688/f1000research.24516.2

**Published:** 2021-10-22

**Authors:** Concetta Brugaletta, Karine Le Roch, Jennifer Saxton, Cécile Bizouerne, Marie McGrath, Marko Kerac

**Affiliations:** 1 Gastrointestinal Physiology Unit, University College London Hospitals NHS Trust, London, England, NW12BW, UK; 2 Mental Health and Care Practices Department, Action Contre la Faim, 75017 Paris, France; 3 Independent, England, UK; 4 Emergency Nutrition Network, Kidlington, England, OX5 2DN, UK; 5 Department of Population Health, London School of Hygiene & Tropical Medicine, London, England, WC1E 7HT, UK

**Keywords:** Breastfeeding, Assessment Tools, Infants

## Abstract

**Background:** Many small and malnourished infants under 6 months of age have problems with breastfeeding and restoring effective exclusive breastfeeding is a common treatment goal. Assessment is a critical first step of case management, but most malnutrition guidelines do not specify how best to do this. We aimed to identify breastfeeding assessment tools for use in assessing at-risk and malnourished infants in resource-poor settings.

**Methods**: We systematically searched: Medline and Embase; Web of Knowledge; Cochrane Reviews; Eldis and Google Scholar databases. Also the World Health Organization (WHO), United Nations International Children’s Emergency Fund (UNICEF), CAse REport guidelines, Emergency Nutrition Network, and Field Exchange websites. Assessment tool content was analysed using a framework describing breastfeeding ‘domains’ (baby’s behaviour; mother’s behaviour; position; latching; effective feeding; breast health; baby’s health; mother’s view of  feed; number, timing and length of feeds).

**Results**: We identified 29 breastfeeding assessment tools and 45 validation studies. Eight tools had not been validated. Evidence underpinning most tools was low quality and mainly from high-income countries and hospital settings. The most comprehensive tools were the Breastfeeding, Evaluation and Education Tool, UNICEF Baby-Friendly Hospital Initiative tools and CARE training package. The tool with the strongest evidence was the WHO/UNICEF B-R-E-A-S-T-Feed Observation Form.

**Conclusions**: Despite many possible tools, there is currently no one gold standard. For assessing malnourished infants in resource-poor settings, UNICEF Baby-Friendly Hospital Initiative tools, Module IFE and the WHO/UNICEF B-R-E-A-S-T-Feed Observation Form are the best available tools but could be improved by adding questions from other tools. Allowing for context, one tool for rapid community-based assessment plus a more detailed one for clinic/hospital assessment might help optimally identify breastfeeding problems and the support required. Further research is important to refine existing tools and develop new ones. Rigorous testing, especially against outcomes such as breastfeeding status and growth, is key.

## Introduction

Protecting breastfeeding has been described as the single most effective child survival intervention ( UNICEF, 2009; WHO, 2007). It also plays a key role in reducing the global burden of undernutrition ( The Lancet Series, 2008) and is one of 13 priority interventions highlighted by the international ‘Scaling Up Nutrition’ movement ( SUN, 2010). Despite this, suboptimal breastfeeding practices are common, accounting for significant morbidity and 804,000 deaths per year - 11.6% of all deaths in children aged under 5 years worldwide ( Black *et al.,* 2013). The greatest burden of mortality and morbidity is in low income countries as defined by the World Bank ( Fantom & Serajuddin, 2016). High background mortality and high rates of undernutrition and communicable disease all make the protective effects of breastfeeding critical. With collapses in infrastructure and normal societal networks, emergency affected populations are particularly vulnerable if breastfeeding is not supported and problems are not quickly identified and addressed. A group particularly higher risk of mortality and morbidity are the small and nutritionally at risk infants under six months of age compared to the infant that achieve optimal growth. At a population level, small and nutritionally at risk children are those identified as wasted, stunted and underweight and a combination of these ( ENN/LSHTM, 2021).

Whilst the importance of breastfeeding is widely recognised, supporting it can be challenging. Under the overall heading of ‘Promoting proper feeding for infants and young children’, the World Health Organization (WHO) lists several areas of work including: the Baby-Friendly Hospital Initiative (BFHI) ( WHO/UNICEF, 2009a); promotion of exclusive breastfeeding; and the International Code of Marketing of Breast-milk substitutes. These initiatives are aimed at population level breastfeeding support; there is good evidence of their effectiveness ( Beake *et al.,* 2012). More challenging is how to help those who fall through these population ‘safety nets’; when an individual mother-infant pair presents with an established problem. Managing very small infants, those with growth failure and other high-risk characteristics is particularly complex. Breastfeeding problems are common in this group but there are many other potential underlying causes and contributory factors ( Goh *et al.,* 2016). Breastfeeding problems may be a primary cause or secondary to other causes. There is also a wide and complex spectrum of breastfeeding problems ranging from a simple positioning difficulty leading to insufficient milk intake, milk insufficiency perception, early complementary feeding introduction, to secondary milk insufficiency due to maternal depression, due in turn to lack of social support at home ( Amir & Ingram, 2008; Moore *et al.,* 2012; Pannu *et al.,* 2011; WHO/UNICEF, 1994).

This review arose from a project exploring the Management of (Nutritionally) At-risk Mothers and Infants aged under 6 months (MAMI) Project ( ENN/UCL/ACF, 2010b). The goal of the original MAMI Project was to investigate the management of malnourished infants under six months of age in resource-poor and humanitarian settings, and to contribute to evidence-based, better practice guidelines to improve practice. The project identified that the burden of infant less than 6 months’ undernutrition is significant: worldwide, 3.8 million infants are severely wasted; 4.7 million are moderately wasted ( Kerac *et al.,* 2011). Since breastfeeding difficulties are associated with undernutrition ( Gagliardi *et al.,* 2012; Gribble *et al.,* 2011) ( Gribble *et al.,* 2011) and exclusive breastfeeding in infants under 6 months, a common treatment goal ( ENN/UCL/ACF, 2010a), the report also examined breastfeeding assessment as part of overall infant assessment. It found no ‘gold-standard’ breastfeeding assessment tool that catered for inpatient and community settings. This is a critical gap; correct ‘diagnosis’ of a breastfeeding problem is vital to inform appropriate support and treatment. Building upon and updating the work of the MAMI Project, this current review thus aims to: a) identify and profile currently available breastfeeding assessment tools; b) discuss their potential application for assessing at risk and malnourished infants under 6 months (i.e. to determine the link between breastfeeding problems and malnutrition in a particular individual; to describe the nature of that breastfeeding problem). Informed assessment is critical to targeted intervention of support.

## Methods

Breastfeeding assessment tools were defined as: documented guidance for clinicians, nurses, midwives, community health workers and carers on how to observe and/or assess the breastfeeding outcomes. These could take the form of checklists, questionnaires, algorithms, indices, history taking forms or listing of the specific aspects of breastfeeding that should be assessed. 

***Inclusion criteria:*** We included articles that: tested or used breastfeeding assessment tools; integrated at least one clinically relevant maternal or child outcome (e.g. duration of breastfeeding, infant weight gain); reported on tool performance. Articles describing complex interventions that included breastfeeding support could only be included if it was clear which tool had been used, and if breastfeeding assessment had been explicitly mentioned in the intervention description. There were no study design restrictions.

***Exclusion criteria:*** Tools that focused just on artificial feeding (i.e. use of a breastmilk substitute) or that were designed for women after breast augmentation/reduction surgery were not considered in this review. Also excluded were tools that involved complex and expensive technology that are not designed for routine clinical use in resource poor settings (e.g. those using electromyographic methods; direct measurements of breastmilk composition; web-based tools; software to measure sucking strength/effectiveness; ultrasound measures of milk removal/swallowing). Tools that focused on wider breastfeeding support (e.g. employer support) rather than the actual process of breastfeeding were also excluded as were those focused solely on change in health worker knowledge, attitude or practice as an outcome. The literature search was restricted to English language articles with human subjects.

***Databases and search terms:*** Articles were identified by searching electronic database Medline and Embase via Ovid interface (full search strategy is free available at *http://www.doi.org/10.17037/DATA.00001881* in *Extended data* ( Kerac *et al.,* 2020)). Key words and MeSH terms were selected by the review on The Lancet Breastfeeding Series ( The Lancet Series, 2016) and a recent similar review on feeding assessment tools ( Howe *et al.,* 2008). We also included hand search papers form grey literature, WHO and ENN websites. Searches were finalised in March 2018. This updated an earlier search done as part of the original MAMI project performed on PubMed, Web of Knowledge, Cochrane Review, Eldis and Google scholar databases which concluded in November 2013. In that original search, highly relevant journals were also searched directly: Maternal and Child Nutrition, International Breastfeeding Journal, Journal of Human Lactation, and BMC Family Practice. Reference lists and the ‘related articles’ were used to identify further articles. A standard two-stage search strategy was used: initial screening of titles and abstracts by 3 authors (C.B, K.L.R. and M.K.); detailed review of full articles secondly (C.B, K.L.R. and M.K.). Since tools were few but varied, risk of bias was not formally scored for each individual study but is discussed under ‘limitations’ for studies as a whole.

### Description of the tools

To understand and characterise the tools we also examined:

### Tool coverage of breastfeeding ‘domains’

There are several aspects or ‘domains’ of breastfeeding. Knowing which are affected helps guide appropriate subsequent treatment. We used an established framework ( Moran *et al.,* 2000) to characterise which aspects of breastfeeding the assessment tools assessed. These included: baby’s behaviour (e.g. alertness to feed), mother’s behaviour (e.g. watches and listens for baby’s cues), positioning (e.g. baby facing mother), attachment (e.g. lower lip turned outward on breast), effective feeding (e.g. sucking, swallowing, jaw movement and signs of milk release), health of the breast (e.g. nipple trauma), health of the baby (e.g. alert), and mother’s experience (e.g. feels strong suction). We added another domain on number, timing and length of feeds. We also noted any other domains identified by individual studies. 

### Evidence underpinning each assessment tool

Studies were grouped according to type of evidence presented. One group looked at prediction of later breastfeeding status. Another assessed test-retest, inter-rater reliability and sensitivity and specificity of tools. A final group of studies focused on assessment tools used to directly improve breastfeeding technique or experience.

## Results

**Figure 1.**
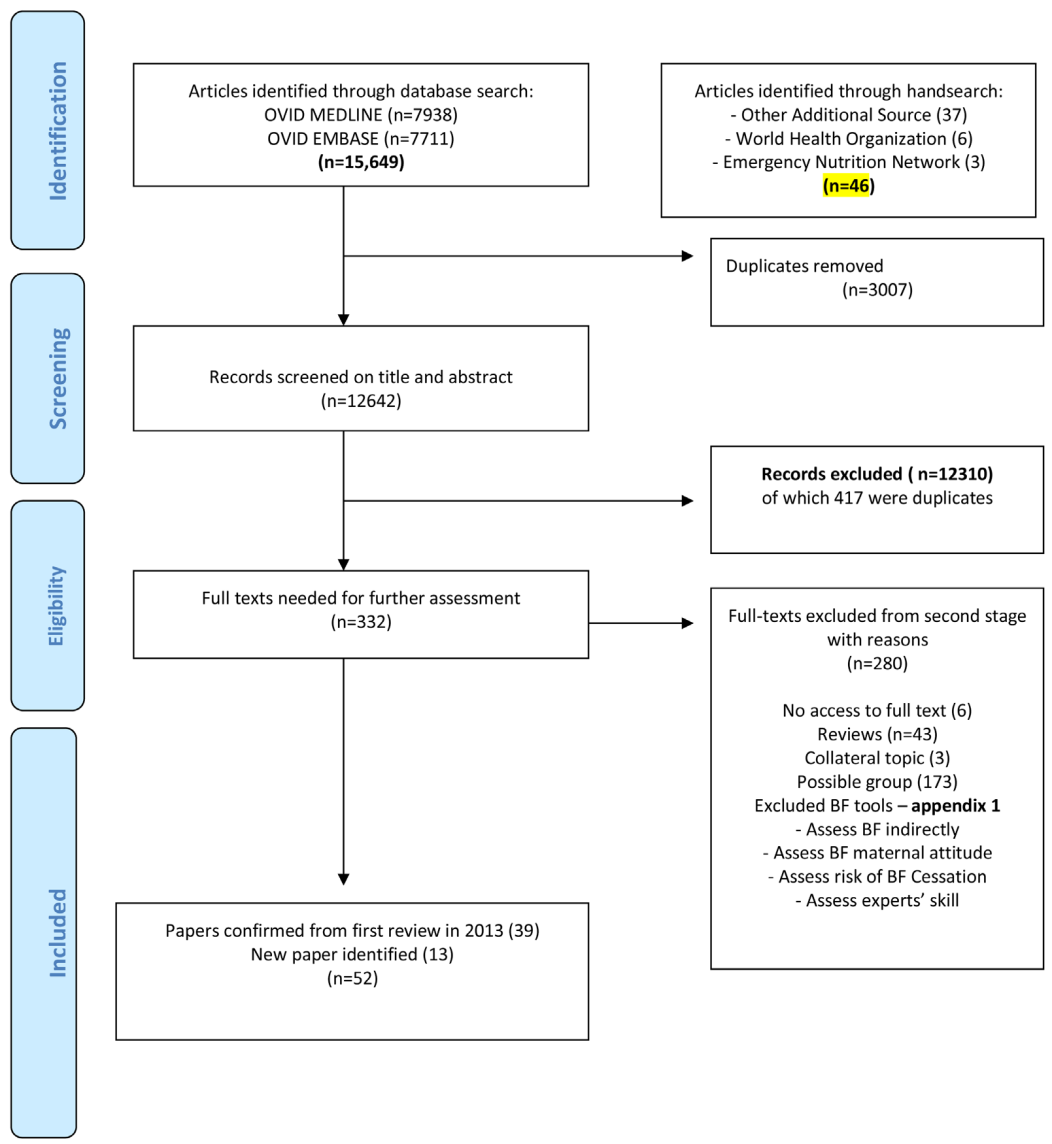
PRIMA Flow diagram: Systematic search for BreastfFeeding Assessment Tools Review. Preferred Reporting items for Systematic Reviews and Meta-Analyses (PRISMA) diagram of literature search results. Diagram retrieved from: http://prisma-statement.org/PRISMAStatement/FlowDiagram.aspx.

From a total of 15,649 titles and abstracts screened, a final count of 52 papers describing 29 distinct breastfeeding assessment tools were identified ( Figure 1).

### Final selection of tools

Details of the 29 tools identified are summarised in Table 1.

**Table 1.** Description of breastfeeding (BF) assessment tools.

Tool name	Author(s) & date	Country of origin	Setting of design	Tool description
WHO/UNICEF Baby-Friendly Hospital Initiative (BFHI) - UNICEF Breastfeeding Assessment Form WHO Breastfeed Observation Job Aid	( UNICEF, 2010) WHO/UNICEF, 2009b ( WHO/UNICEF, 2009b)	Worldwide	Hospital and Community	**Breastfeeding Assessment Form:** 14 questions and observations where answers indicate effective feeding or a problem. Items cover baby’s health, urine and stools, behaviour during/after feeds, frequency of feeds, mother’s behaviour during a feed, breast condition, use of dummies, nipple shields, and formula. If problems are identified, observe a full breastfeed, using the observation aid.
				**Additional training material:** comfort of the mother (2), help with positioning (4), how to support breasts to facilitate attachment (5), signs of good/poor attachment (as per observation aid), releasing suction before removing child from breast. Special guidance for low birth weight: helpful breastfeeding positions, expressing milk. Weight gain and urine output differentiate ‘perceived’ and ‘real’ insufficient milk. **Breastfeeding Observation Job Aid**: 42 items/5 scales; signs of BF going well versus possible difficulty: general (mother and baby); breasts; baby’s position; baby’s attachment; suckling.
Breast-feeding Assessment Score (BAS)	( Hall *et al.,* 2002)	USA	Hospital	5 variables assessed: mother’s age, previous breastfeeding experience, lactating problems, breastfeeding interval, bottles of formula. Extra variables: breast surgery, maternal hypertension, vacuum vaginal delivery. To identify infants at risk for early cessation of breastfeeding before initial discharge from hospital.
Breastfeeding Evaluation & Education Tool (BEET)	( Tobin, 1996)	USA	Not specified	8 sub-scales: feedings, positioning, latch, suck, milk flow, intake, output, weight gain. To help parents observing and assessing the evolution of breastfeeding and seek guidance from health care providers upon necessity.
CARE training package: Breastfeeding and Complementary Feeding Basics	( CARE, 2004)	LMICs	Community humanitarian settings	Training materials include handouts and counselling cards on: Signs of good positioning (4 items) and attachment (5 items, 1 illustration) and effective suckling (5 points); recommendations on optimal breastfeeding practices focusing on mother’s behaviour; 3 common breast conditions (including photos); perceived insufficient milk supply; 11 ‘special situations’ including malnourished and stressed mothers, baby refusal to feed. Prevention and solutions are given.
Checklist from 'breastfeeding and the use of pacifiers'	( Righard & Alade, 1992; Righard & Alade, 1997)	Sweden	Hospital	16 observations to determine early breastfeeding cessation and correct vs incorrect sucking techniques: breast offering (3 items), sucking at the breast (9 items), after feeding (2 items), and conclusions (2 items).
Essential Nutrition Action Messages (Breastfeeding guidance booklet)	( Guyon & Qinn, 2011; Guyon *et al.,* 2009)	LMICs	Specifies multiple settings for use	Illustration and recommendations to ensure optimal breastfeeding. Illustration 8 on correct positioning: 9 guidance items + 3 pictures. Illustration 9 focuses on proper attachment: 4 signs and 5 signs of efficient suckling + 1 picture. There is also illustration 10 for three different breastfeeding positions and attachment, with pictures.
History Taking Form from ‘Functional assessment of infant breastfeeding patterns’	( Walker, 1989)	USA	Not specified	A sample feeding assessment with rationale that covers: general physical condition and body tone of baby; with a digital check of infant sucking ability, breast assessment (e.g. look for engorgement), nipple assessment (e.g. flat nipple), position of mother and baby whilst nursing, latch on, sucking pattern/sound, and maternal impression of the feed. It is part of a general assessment of normal and problematic situations that include a baby’s feeding history and the mother’s history on some physical aspects before and after pregnancy.
Hands off technique	( Ingram *et al.,* 2002)	UK	Hospital	8 guidelines to teach mothers in 'hands off' way to position and attach baby. Includes leaflet with pictures and explanations about breastfeeding
Integrated Management of Childhood Illness (IMCI) algorithm *adapted in* *Bangladesh*	( Mannan *et al.,* 2008)	Bangladesh	Community	History taking and observation classifies children as: ‘not able to feed’ (very severe disease), ‘feeding problem’, and ‘no feeding problem’. Four questions ask about: any breastfeeding difficulty, newborn feeding ability, feeding frequency and supplementary foods. Observations are made of a five-minute breastfeed including: four signs of improper attachment, four signs of improper positioning, and one sign of sucking effectiveness (‘slow, deep sucking with occasional pausing’).
Integrated Management of Neonatal and Childhood Illness (IMNCI) algorithm	( Dongre *et al.,* 2010) ( WHO/UNICEF/National-Rural-Health-Mission, 2009)	India	Health center	Uses four signs of good positioning and four signs of good attachment. Observers recorded ‘yes’ or ‘no’ for each sign. ‘Take action cards’ are used to resolve breastfeeding problems.
From ‘Indicators of effective breastfeeding and estimates of breast milk intake’	( Riordan *et al.,* 2005)	USA	Not specified	Breastfeed is scored 0-2 (0=absent behaviour, 1=problematic, 2=no problem): 1) Rooting 2) length of time before latch on 3) latch-on 4) suckle 5) observable swallowing 6) audible swallowing.
Infant Breastfeeding Assessment Tool (IBFAT)	( Matthews, 1988; Matthews, 1998)	Canada	Hospital	6 items measure four infant behaviours: readiness to feed, rooting, fixing & sucking. Two non-scoring items: infant state & maternal satisfaction with breastfeeding
Infant Feeding in Emergencies (IFE) Module 2: Simple rapid assessment and full assessment	( ENN *et al.,* 2007)	LMICs	Community humanitarian settings	Simple rapid assessment includes: age appropriate feeding, breastfeeding ease, baby's condition. Refer problems for full breastfeeding observation: attachment, suckling, mother’s confidence, feed end. Listen/learn from mother about feeding practices/beliefs/worries. Observe artificial feed if relevant. Breastfeeding assessment based on WHO 40 hour breastfeeding counselling course (2004)
LATCH Assessment	( Jensen *et al.,* 1994a; Jensen, 1994b)	USA	Not stated	5 items: Latch; Audible swallowing; Type of nipple; Comfort of mother's breasts/ nipples; Help needed to hold baby to breast. Mother’s ability to attach her baby properly to the breast and observe her infant sucking
From ‘Lactating on and suckling of the healthy term neonate’	( Cadwell, 2007)	USA	Hospital	Clinical strategies for systematic assessment of breastfeeding: 1) Pre-feeding behaviours (rooting, increased alertness, brings hand to mouth, sucking, mouthing) and one picture pre-latch-on 2) Six aspects of latch-on and suckling dynamic 3) three aspects of milk transfer from mother to infant 4) one aspect of mothers comfort during/between feedings 5) one aspect of infant signs of satiety
Mother-Baby Assessment (MBA)	( Mulford, 1992)	USA	Hospital	5 steps in breastfeeding are assessed for both the mother and the infant: signalling, positioning, fixing, milk transfer, ending. A score out of ten rates mother’s and baby’s efforts to breastfeed and the progress of both partners. Tool items based on positioning, fixing & milk transfer items from published work describing common features of effective breastfeeding.
Mother-infant breastfeeding assessment tool	( Johnson *et al.,* 1999)	USA	Community	Mother and infant scored on 8 items to indicate risk of breastfeeding failure: latch, suck, nipple type, frequency of nursing/wet nappies, previous success with breastfeeding, supportive partner.
Mother-Infant Breastfeeding Progress Tool (MIBPT)	( Johnson *et al.,* 2007)	USA	Hospital	8 items observe breastfeeding progress in the dyad: responsiveness to feeding cues, timing of feeds, nutritive suckling, positioning/ lactating factors, nipple trauma, infant behaviour state and mother/parent response to the infant.
Neonatal Oral-Motor Assessment Scale (NOMAS)	( Palmer *et al.,* 1993)	USA	Hospital	28 items: nutritive/ non-nutritive sucking. Outcomes: normal, disorganised or dysfunctional feeding. Latter two feeding types are graded by severity (mild, moderate, severe)
Preterm Infant Breastfeeding Behaviour Scale (PIBBS)	( Nyqvist *et al.,* 1996)	Sweden	Hospital	Observations of developmental process of sucking during breastfeeding for preterm infants on: rooting, areolar grasp, duration of latch, sucking, longest sucking burst, swallowing.
From ‘Sucking technique and its effect on success of breastfeeding’	( Righard & Alade, 1992)	Sweden	Hospital	Focuses on assessment of sucking technique as correct or incorrect. Correct defined as infant has wide-open mouth, tongue under areola, milk being expressed in slow deep sucks. Incorrect defined as sucks at the nipple as if it is a teat. Visual tools.
Systematic Assessment of the Infant at Breast (SAIB)	( Shrago & Bocar, 1990)	USA	Hospital	Observation of: alignment, areolar grasp, areolar compression, audible swallowing. No scoring system. Assess effective breastfeeding and milk transfer.
VIA Christi Breastfeeding assessment	( Riordan, 1999)	USA	Not stated	Breastfeed is scored 0-2 (0=absent behaviour, 1=problematic, 2=no problem): 1) latch on 2) time before latch on and suckle 3) suckling 4) degree of swallowing 5) mother’s evaluation. Overall Scores 0-2 = high risk, return visit/call within 24 hours (automatic high risk if >10% birth weight lost or mother had breast surgery); 3–6 = medium risk, refer to public health nursing, visit within 3 days; 7–10 = low risk, routine calls/visits. to assess excessively sleepy baby following high dose of labour analgesia.
WHO/UNICEF B-R-E-A-S-T-Feed Observation Form	( WHO/UNICEF, 1994)	Worldwide	Community	27 items/6 scales: signs that breastfeeding is going well versus possible difficulty: body position, responses, emotional bonding, anatomy, suckling, time suckling.
Neonatal Eating Assessment tool (NeoEAT)	( Pados *et al.,* 2016; Pados *et al.,* 2018)	USA	Hospital	Screener version (10 questions) is intended for clinical screening of infants to identify whether further specialist assessment is needed. Full version is for specialized assessment for use in feeding and dysphagia specialty clinic and research: NeoEAT Breastfeeding (72 items), NeoEAT Bottle Feeding (74 items), and NeoEAT Breastfeeding and Bottle Feeding (89 items). It can be used by parent or health workers to assess breastfeeding in infants less than 7 months.
Early Feeding Skills Assessment (EFS)	( Thoyre *et al.,* 2018)	USA	Hospital	The checklist allows health workers to assess preterm infant readiness for breastfeeding. This helps profiling the infant's developmental stage regarding specific feeding skills: abilities to remain engaged in feeding, organize oral-motor functioning, coordinate swallowing with breathing, and maintain physiologic stability to decide which help offer.
Preterm Oral Feeding Readiness Assessment Scale (POFRAS)	( Fujinaga *et al.,* 2013)	Brazil	Hospital	18 items scale to assist health professionals to initiate preterm feeding in view of promoting safe and objective breastfeeding. Focus on baby's ability and readiness to suckle well.
Bristol Breastfeeding Assessment Tool (BBAT)	( Ingram *et al.,* 2015)	UK	Hospital	4-item tool: position, attachment, sucking and swallowing to improve targeting positioning and attachment advice. Attribution of a 0 to 2 score: 0 poor - 2 good or no need advice. It can be useful on tongue-tied infant.
Lactation history and risk assessment form	( Riordan, 1989)	USA	Hospital	4-items form to take lactation history and evaluate breast and nipples to carry out an appropriate risk assessment: feeding choice, physical exam, history including baby weight gain, risk factors.

Exclusions and reason for those are presented in web-appendix ( *Extended data* ( Kerac *et al.,* 2020)). We were unable to get sufficient information about two tools: The LAT ^TM^ ( Cadwell *et al.,* 2004) and the Prague Newborn Behaviour Description Technique ( Sulcova & Tisanska, 1994) so we could not include them in the final review.

### Context

Of the 29 tools identified: 22 (76%) were developed in high-income countries and used in 31 studies carried out in high-income countries, six (21%) tools were developed in low and middle-income countries and one (3%) was developed worldwide. Sixteen tools (55%) were developed for hospital settings. Of these, 24 (83%) tools were designed and/or tested for use in infants less than 6 months with breastfeeding problems; none of these were specifically designed for or tested on at risk and malnourished infants less than 6 months.

### Coverage of breastfeeding domains

Table 2 shows that most tools covered a number of different domains but only one, the Breastfeeding Evaluation and Education Tool ( Tobin, 1996), covered them all.

**Table 2.** Breastfeeding domains of primary interest covered by assessment tools (domain based on Moran *et al.,* 2000.

Assessment Tool	Baby's Behaviour	Mother's Behaviour	Position	Lactating	Effective Feeding	Breast health	Baby’s health	Mother's view of the feed	Number, timing, length of feeds	Other
WHO/UNICEF Baby Friendly Hospital Initiative: UNICEF Breastfeeding assessment form & WHO/UNICEF Breastfeed Observation Job Aid ( UNICEF, 2010) ( WHO/UNICEF, 2009b)	□	□	□	□	□	□	□	□		Positions if low birth weight, insufficient milk, mother’s health, formula, dummies
Breast-feeding Assessment Score ( Hall *et al.,* 2002)				□						Breast surgery, maternal hypertension and delivery type
Breastfeeding evaluation and education tool ( Tobin, 1996)	□	□	□	□	□	□	□	□	□	Signs of milk transfer in mother (e.g. uterine cramps)
CARE training package: Breastfeeding and Complementary Feeding Basics ( CARE, 2004)	□	□	□	□	□	□	□		□	Positions for low birth weight babies, perceived insufficient milk, mother’s health
Checklist from “Breastfeeding and the use of pacifiers” ( Righard & Alade, 1997)	□	□	□	□	□	□		□	□	
Essential Nutrition Action Messages ( Guyon *et al.,* 2009)			□	□	□				□	
History taking form from “Functional assessment of infant breastfeeding patterns” ( Walker, 1989)	□		□	□	□	□	□	□	□	Digital check of sucking ability
Hands off technique ( Ingram *et al.,* 2002)	□	□	□	□	□					
IMCI algorithm ( Mannan *et al.,* 2008)			□	□	□			□	□	Supplementary food
IMNCI algorithm ( Dongre *et al.,* 2010)			□	□						
From “Indicators of effective breastfeeding and estimates of breast milk intake” ( Riordan *et al.,* 2005)	□		□	□	□					Observable and audible swallowing
IBFAT Infant Breastfeeding Assessment Tool ___( Matthews, 1988)	□			□	□			□		Baby’s readiness to feed
Infant Feeding in Emergencies Module 2: Simple rapid assessment and full assessment ( ENN *et al.,* 2007)	□	□		□	□	□	□	□	□	Other food/liquid, feed end, pacifiers, mother’s beliefs and worries
LATCH Assessment ( Jensen *et al.,* 1994a)	□		□	□	□	□		□	□	Need assistance to breastfeed
“Latching on and suckling of the healthy term neonate” ( Cadwell, 2007)	□	□	□	□	□	□				Mother’s comfort level; pre-feeding behaviors
MBA Mother-Baby Assessment ( Mulford, 1992)	□	□	□	□	□	□		□		Pre-feeding behaviors
Mother-infant Breastfeeding Assessment Tool ( Johnson *et al.,* 1999)				□	□	□			□	Previous feeding experience, partner support
MIBPT - Mother Infant Breastfeeding Progress Tool ( Johnson *et al.,* 2007)		□	□	□	□	□		□	□	
NOMAS ( Palmer *et al.,* 1993)				□	□					
PIBBS ( Nyqvist *et al.,* 1996)	□			□	□				□	
SAIB ( Shrago & Bocar, 1990)	□	□	□	□	□					Pre-feeding behaviors
“Sucking technique and its effect on success of breastfeeding” ( Righard & Alade, 1992)				□	□					Visual tool - pictures available
VIA Christi Breastfeeding assessment (unpublished)				□	□			□		>10% birth weight lost
WHO/UNICEF B-R-E-A-S-T-Feed Observation Form ( WHO/UNICEF, 1994)	□	□	□	□	□	□			□	
NeoEAT - Neonatal Eating Assessment Tool ( Pados *et al.,* 2017; Pados *et al.,* 2018)	□		□	□	□		□			Evaluate oral pharingo esophageal function, gastrointestinal function
Preterm oral Feeding Readiness Assessment Scale ( Fujinaga *et al.,* 2013)	□		□	□	□					
EFS - Early Feeding Skills assessment ( Thoyre *et al.,* 2005)	□		□	□	□				□	Ability to remain engaged in feeding. Ability to Organize Oral-Motor Functioning including swallowing
BBAT Bristol Breastfeeding Assessment Tool ( Ingram *et al.,* 2015)	□		□	□	□					Can be used also on tongue-tied infant
Lactation history and risk assessment form (( Riordan, 1989)		□				□	□			Estimate risk of developing a problem before giving birth

Other tools covering a wide range of domains were the Baby-Friendly Hospital Initiative (BFHI) guidelines ( UNICEF, 2010; WHO/UNICEF, 2009a) and the CAse REport guidelines (CARE guidelines) ( CARE, 2004). The BFHI and CARE guidelines also highlighted other items that could be useful for future testing: positions for low birth weight babies, differentiating between ‘perceived’ and ‘real’ milk insufficiency, mother’s health, and the use of BMS and dummies/pacifiers. The World Health Organization/United Nations International Children’s Emergency Fund (WHO/UNICEF) B-R-E-A-S-T-Feed Observation Form covered seven domains, missing out ‘health of the baby’ and ‘mother’s view of the feed’ ( WHO/UNICEF, 1994). Additional domains identified by other tools included: mother’s comfort level, previous breastfeeding experience, other foods/liquids being given to the baby, loss of >10% of birth weight, hypertension and delivery type ( Darmstadt *et al.,* 2009; Dongre *et al.,* 2010; Hall *et al.,* 2002; Mannan *et al.,* 2008; Milligan *et al.,* 1996; Palmer *et al.,* 1993). 

### Ability of tools to predict breastfeeding outcomes

In total, 12 (41%) tools had been tested for their ability to predict breastfeeding outcomes ( Table 3).

**Table 3.** Studies relating to tool validity assessing breastfeeding-related outcomes.

Assessment Tool	Author & date	Country & setting	Sample	Study design	Outcomes	Findings	Remarks
WHO/UNICEF Baby- Friendly Hospital Initiative: UNICEF Breastfeeding assessment form & WHO/UNICEF Breastfeed Observation Job Aid	( Geddes, 2012)	UK, Community	Mothers and babies 5-12 days after delivery; N not given	Time trend analysis: from intervention baseline to 3 years, quarterly data points.	% of women breastfeeding at 6-8 weeks. Intervention: home-visits to resolve feeding problems using breastfeeding observation aid	Breastfeeding at 6-8 weeks was 60.5% at baseline, increased to 61.6%, then steadily increased each quarter to 68.9% in the third quarter post intervention	No control group. Source of regional breastfeeding prevalence data not clear. Difficult to extract influence of breastfeeding assessment tool
WHO/UNICEF Baby- Friendly Hospital Initiative: UNICEF Breastfeeding assessment form & WHO/UNICEF Breastfeed Observation Job Aid	( Ingram *et al.,* 2011)	UK, Community	Bristol-born children at 8 weeks of age; N not given	Time trend analysis: breastfeeding rates pre/post BFHI training	Annual breastfeeding rates 2006-9 (routine 8 week check); staff knowledge, attitudes, confidence and self- efficacy	Babies born in 2009 were 1.57 times more likely to be breastfed, and 1.46 times more likely to be exclusively breastfed at 8 weeks.	No control group. Difficult to extract influence of breastfeeding assessment tool
Breastfeeding Assessment Score (BAS)	( Hall *et al.,* 2002)	USA, hospital	N=1108 mothers and infants; mean age=40 hours	Observational	Breastfeeding cessation 7-10 days postpartum	10.5% of mothers reported stopping breastfeeding; all tool items significantly predicted breastfeeding cessation.	No information on maternal or infant health indicators
Breastfeeding Assessment Score (BAS)	( Gianni *et al.,* 2006)	Italy, hospital	N=175 mothers of healthy exclusively breastfed infants; birth weight ≥2500g, gestational age 37-42 weeks	Observational	Breastfeeding cessation, introduction of complementary feeding, continued exclusive breastfeeding at 1 month	Women exclusively breastfeeding at 1 month had significantly lower baseline BAS scores than women not exclusively breastfeeding. Lactating problems and no prior breastfeeding success was negatively associated with breastfeeding duration.	
Breastfeeding Assessment Score (BAS)	( Mercer *et al.,* 2010)	USA, hospital	N=1182 mother-child pairs	Observational	Breastfeeding 7-10 days postpartum	Maternal age, previous breastfeeding experience, lactating difficulty, breastfeeding interval, number of bottles and total BAS score were significantly predictive of breastfeeding cessation 7-10 days postpartum	Many participant exclusions e.g. children <24 hours old, women <18, depression Covariates: Adjusted for between hospital differences
Breastfeeding Assessment Score (BAS)	( Zobbi *et al.,* 2011)	Italy, hospital	N=380 women	Observational	Sensitivity and specificity of BAS	Reduced BAS (5 items) and adapted cut off for predicted breastfeeding cessation from 8 to 9 optimised BAS sensitivity: 77.9%, and specificity=56.9.	Excluded non-Italian mothers, twin births and those born <26 weeks. Covariates: Epidural, gluconate, dummy use, antenatal care
Checklist from breastfeeding and the use of pacifiers	( Righard & Alade, 1997)	Sweden, Hospital	N=82 exclusively breastfeeding mothers with intention to breastfeed ≥6 months. Infants had normal deliveries/ birth weights, 4-5 days postpartum	Observational	Breastfeeding rate & pacifier use (hours/day) at 2 weeks, 1, 2, 3 & 4 months of pacifier and non-pacifier users and children with correct/ incorrect sucking technique	Pacifier users with correct sucking technique had higher levels of breastfeeding at 4 months than Incorrect sucking group. Pacifier users had significantly lower breastfeeding rates than non-users. No difference in breastfeeding amongst non-pacifier users with correct/ incorrect sucking technique	Incorrect sucking technique may not be improved if pacifiers are used
Essential Nutrition Actions	( Guyon *et al.,* 2009)	Madagascar, Clinic and community	Baseline n=1200, Endline n=1760 children <2	Baseline/ Endline intervention survey	Infant and young child feeding indicators, feeding during illness, deworming, maternal diet and health	Exclusive breastfeeding <6 months increased from 32% to 68%	No control group; difficult to extract influence of breastfeeding assessment tool
Hands Off Technique	( Ingram *et al.,* 2002)	UK, Hospital	N=395 mothers who were breastfeeding on discharge	Observational	Breastfeeding (any and exclusive) 2 & 6 weeks postpartum	High breastfeeding technique score was associated with breastfeeding at 6 weeks.	Short, pragmatic training for midwives to teach good breastfeeding technique Covariates: Use of dummy, partner support, milk production, nipple pain
Hands Off Technique	( Wallace *et al.,* 2006)	UK, Hospital	N=245 midwives randomized to ‘hands off’ protocol or refresher standard care; n=370 women randomized to midwives postpartum	RCT	Duration of breastfeeding (exclusive and any breast milk) at 6 and 17 weeks postpartum.	No significant differences between groups on any or exclusive breastfeeding at 6 or 17 weeks, or in reported breastfeeding problems	Study was statistically underpowered to detect an effect; authors suggest initial feeding advice may be best as hands on, with ‘hands off introduced later Covariates: Hospital, delivery type, maternal age, prior feeding experience, midwife grade
IMCI algorithm	( Mannan *et al.,* 2008)	Bangladesh, Community	N=3495 neonates	Observational	Breastfeeding problems 1-7 days postpartum	Women only receiving a postnatal visit at 6-7 days were 7.66 times more likely to have breastfeeding difficulties than those receiving early and late postnatal visit (1-3 days and 6-7 days)	Coverage 63%-77%; home visits had structured assessment of breastfeed and corrective advice Covariates: Prim-parity, prematurity, low birth weight, pre-lacteals
‘Indicators of effective breastfeeding and estimates of breast milk intake’	( Riordan *et al.,* 2005)	USA, Hospital	N=82 mothers and their term infants	Observational	Significant predictors of human milk intake in children ≤96 hours and >96 hours (through test weighing)	Rooting and observable swallowing were significant predictors of milk intake at ≤96 hours; audible swallowing at >96 hours	Swallowing and rooting in first 3 days, audible swallowing >3 days should be included in breastfeeding assessments of term infants. Covariates: Maternal age, previous feeding experience, delivery type, infant sex, birth weight, gestational age
Infant Breastfeeding Assessment Tool (IBFAT)	( Matthews, 1988)	Canada, hospital	N=60 early neonates with appropriate weight for gestational age	Observational	Breastfeeding status at 4 weeks; inter-rater reliability	IBFAT scores did not predict breastfeeding at 4 weeks. Inter-rater agreement=91%.	Authors: scores may not have predicted breastfeeding due to limited variability (80% were still breastfeeding).
Infant Breastfeeding Assessment Tool (IBFAT)	( Schlomer *et al.,* 1999)	USA, Hospital	N=30; First time breastfeeding mothers of term infants	Observational	Association between maternal satisfaction & breastfeeding problems from 12 hours to 1 week postpartum	Low predictive validity for maternal satisfaction & breastfeeding problems (r=0.379, p=0.163), but IBFAT scores were negatively related to breastfeeding problems (r=-0.49, p=0.06)	Very small sample size and low predictive validity of maternal satisfaction with breastfeeding
LATCH	( Riordan *et al.,* 2001)	USA, Hospital	N=133 mothers of healthy singletons (38- 42 weeks gestational age). Mothers were intending to breastfeed.	Observational: post-partum and followed 6 weeks	Breastfeeding status	Mothers’ breastfeeding at 6 weeks had higher LATCH scores than those who had weaned. Mothers scoring lower on comfort were less likely to be breastfeeding at 6 weeks postpartum.	Query that audible swallow is possible on day 4 of life Covariates: Mother’s age, intended breastfeeding duration & delivery type
LATCH	( Kumar *et al.,* 2006)	USA, Hospital	N=182 (4 days) N=188 (6 weeks) mother-child pairs; healthy term infants	Observational: day 1 till 6 weeks after delivery	Breastfeeding status	Women breastfeeding at 6 weeks had significantly different LATCH scores at 0–48 hours than those not breastfeeding. ROC curve: scores of ≥9 at 16–24 hours linked to a 1.7 times greater chance of breastfeeding at 6 weeks. Nurse/ mothers scores correlated with breastfeeding duration.	
LATCH	( Schlomer *et al.,* 1999)	USA, Hospital	N=30 first time breastfeeding mothers of term infants	Observational: 12 hours and 1 week post- partum	Association between maternal satisfaction and breastfeeding problems	Low predictive validity for maternal satisfaction & breastfeeding problems (r=0.427, p=0.113) but LATCH scores were negatively related to feeding problems (r=-0.50, p=0.057)	Small sample and poor predictive validity re breastfeeding satisfaction
LATCH	( Henderson *et al.,* 2001)	Australia, Hospital	N=160 first-time mothers	RCT: structured one-to-one positioning and attachment education versus usual postnatal care. at 6 weeks and 3 and 6 months postpartum.	Breastfeeding status Nipple pain /trauma and satisfaction with breastfeeding	No difference in breastfeeding rate between groups. Experimental group had less nipple pain on days 2 and 3, but not sustained. Experimental group were less satisfied with breastfeeding using a single but not a multiple item measure	Mixes use of LATCH tool with hands off intervention technique Covariates: No socio- demographic differences between groups
LATCH	( Tornese *et al.,* 2012)	Italy, Hospital	N=299 mother-infant dyads	Observational: day 1 and at discharge from hospital	Non-exclusive breastfeeding at discharge from hospital	LATCH score in the first 24 hours predicted non-exclusive breastfeeding at discharge	Covariates: Caesarean, primiparity, infant phototherapy
Mother-infant breastfeeding assessment tool	( Johnson *et al.,* 1999)	USA, Community	N=981 infants	Observational	Readmission to hospital due to child ill health or feeding problem	Readmission rate higher if no home visit was made to assist with breastfeeding.	Does not test tool reliability or validity, no covariate adjustment
NOMAS	( Bingham *et al.,* 2012)	USA, Hospital	N=51, premature, tube-fed infants	Observational	Ability of NOMAS to predict readiness to move from tube to oral feeding	NOMAS was a poor predictor of feeding outcomes	Covariates: Gestational age at birth, birth weight, Apgar score, days of respiratory support
WHO/UNICEF B-R-E-A-S-T-Feed Observation Form	( De Oliveira *et al.,* 2006)	Brazil, Hospital	N=74 women randomized to 30 minute breastfeed assessment and technique advice session; N=137 standard care	RCT	Exclusive breastfeeding rate and lactation related problems in the first 30 days post- partum	Intervention and control groups had similar rates of EBF at 7 and 30 days postpartum; there were no differences in nipple problems or breast conditions, or quality of breastfeeding technique.	Authors adapted the b-r-e- a-s-t tool. A single input may not be enough to resolve breastfeeding problems
WHO/UNICEF B-R-E-A-S-T-Feed Observation Form	( Goyal *et al.,* 2011)	Libya, Hospital	N=192 mother-child pairs	Observational	Grading of position, attachment and effective suckling	Associated with poor positioning: primiparous women. Poor attachment: primiparous women, cracked nipples, mastitis, preterm and low birth weight. Poor suckling: preterm, low birth weight & early neonatal period	Adapted the b-r-e-a-s-t form to include a grade (poor, average, good) and a score for breast feeding aspects
WHO: Breastfeeding counselling: a training course	( Kronborg & Vaeth, 2009)	Denmark, Home visits	N=570 mother- child pairs, 1 week postpartum. Randomised to health visitor intervention, with classification and correction of breastfeeding technique, or standard care	RCT	Duration of exclusive breastfeeding	Half of women had breastfeeding problems, most commonly: ineffective position and latch. Adjusted analysis: ineffective technique and pacifier use associated with early breastfeeding problems and reduced duration. A single correction not associated with duration or occurrence of problems.	As a single breastfeeding correction was not effective. Authors suggest ongoing support to correct problems may be necessary Covariates: Early feeding problems, education, previous breastfeeding experience, formula supplement within 5 days of birth
WHO/UNICEF B-R-E-A-S-T-Feed Observation Form	( Kronborg *et al.,* 2007)	Denmark, Home visits	N=780 mother-child pairs randomized to intervention (health visitors classified and corrected breastfeeding technique during 1-3 home visits), n=815 to standard care	Cluster RCT	Duration of exclusive breastfeeding and maternal satisfaction with breastfeeding during 6 months of follow-up	Intervention group had 14% lower breastfeeding cessation rate, received their first home visit earlier, had more home visits in total and more practical breastfeeding training within 5 weeks. Feeding frequency was higher, and fewer used pacifiers. Mothers reported more confidence in milk sufficiency	
WHO/UNICEF B-R-E-A-S-T-Feed Observation Form	( Leite *et al.,* 2005)	Brazil, Home visits	N=1003 infants <3000g n=503 intervention: 6 home visits 5-120 days postpartum, n=500 control	RCT	Feeding methods at 4 months	Exclusive breastfeeding was significantly higher in the experimental group than the control (24.7% vs. 19.4%) and showed a 39% increase in any breastfeeding	Difficult to unpick the effect of observing the breastfeed from other activities. Covariates: Socio- demographic and pregnancy variables
WHO/UNICEF B-R-E-A-S-T-Feed Observation Form	( Yalcin & Kuskonmaz, 2011)	Turkey, Hospital	N=82 mothers and children 2 months of age	Observational	Determinants of score on b-r-e-a-s-t assessment	Female babies had better scores. Associated with worse scores: long bouts of crying, sibling history of colic, short duration of night sleeping, regurgitation.	
LATCH	( Lau *et al.,* 2016)	Singapore	N= 907 mothers and children.	Observational within 72 hours postpartum	Evaluation of internal consistency, validity, sensitivity and specificity 5- and 4-item versions of LACTH. Data were filtered: **preterm deliveries were excluded** because of their different suckling patterns. Only 4 or 5 outcomes. The sample were infant with body weight 3.14-0.39 Kg.		The 4-item versions can be considered as routine assessment tool to assist. The **sensitivity** of the tools to correctly identify postanal woman at risk of non- exclusive breastfeeding is satisfactory (cut off point 3.5 and 5.5) the **specificity** is poor. Acceptable **internal** **consistency**.
LATCH	( Kucukoglu & Celebioglu, 2014)	Turkey	N=85 low birth weight (< 2500g) infants and mothers		Effect of an education intervention half an hour per day during the first 5 days of hospitalization.		low internal consistency
Preterm Oral Feeding Readiness Assessment Scale (POFRAS)	( Fujinaga *et al.,* 2013)	Brazil, Hospital	N= 60 preterm infants	Observational	Accuracy, sensitivity and specificity of POFRA cut-off was demonstrated.		
NeoEAT- Breastfeeding	( Pados *et al.,* 2018)	USA	N=402 parents of 7 months old baby	web-based surveys	parents recruited were asked to use the tool to report child BF problems.		good evidence of reliability and content validity scoring 5.1 consistent with recommendation for health- related materials
Bristol Breastfeeding Assessment Tool (BBAT)	( Dolgun *et al.,* 2018)	Turkey, hospital	N=127 mothers of 0-6 months old baby	Observational: 2 pediatric nurses	Tool was translated. Clarity and fluency language were analysed. Current validity with LACTH tool was explored.		The tool could validly measure the intended construct.
Early Feeding skills Assessment Tools (EFSAT)	( Thoyre *et al.,* 2018)	USA, hospital	N=8 cases of 2 months old baby - 142 feeding observed	Observational	Current validity with Infant-Driven Feeding Scale Quality(IDFS- Q) tool, infant birth risk expressed in gestational age (GA) and infant maturity expressed in post menstrual age (PMA)		Correlation with IDFS-q tool. Later gestational age associated with higher EFS score. Advanced PMA was associated with higher feed engagement subscale score.

The present studies either tested the tools or tested the intervention or tested both. The tools with the most studies testing their ability to predict breastfeeding outcomes during an intervention study were the LATCH (n=5), the WHO/UNICEF B-R-E-A-S-T-Feed observation form (n=6) and the BAS tool (n=4). The BAS was consistently predictive in all studies, although as shown in Table 2, it covers the least number of breastfeeding domains. There were mixed findings for the LATCH tool: three studies observed positive findings, and two reported limited ability of the tool to predict breastfeeding outcomes. The WHO/UNICEF B-R-E-A-S-T-Feed Observation Form was predictive of breastfeeding outcomes in three studies, but was not predictive of exclusive breastfeeding in a fourth study. Two further studies described the determinants of poor scores on the WHO/UNICEF B-R-E-A-S-T tool including repeated crying, colic history, shorter sleeping episodes and regurgitation ( Yalcin & Kuskonmaz, 2011), and primiparity, cracked nipples, mastitis, preterm and low birth weight babies and poor suckling ( Goyal *et al.,* 2011).

### Evidence underpinning the tools

The extent of tool testing varied substantially; 8 tools had no validation studies: Infant Feeding in Emergencies (IFE) Module 2 ( ENN *et al.,* 2007), Breastfeeding Evaluation and Education Tool ( Tobin, 1996), Systematic Assessment of the Infant at the Breast ( Shrago & Bocar, 1990), CARE guidelines ( CARE, 2004), Via Christi, and tools identified by Walker ( Walker, 1989), ( Cadwell, 2007) and Righard & Alade, 1992 ( Righard & Alade, 1992). Of the remaining 21 tools, we identified 45 validation studies. Of these, 32 were observational studies; 6 were randomised or cluster randomised controlled trials, two reported time trends; and 1 reported intervention baseline and endline data without a control group.

The BAS tool had four validation studies, all of which show positive results for the tool, in terms of ability to identify those at risk of breastfeeding cessation, and moderate sensitivity and specificity ( Gianni *et al.,* 2006; Hall *et al.,* 2002; Mercer *et al.,* 2010; Zobbi *et al.,* 2011). The evidence to support the use of the Essential Nutrition Actions Framework tool is weak in terms of validation (i.e. no control group; not clear if the tool was routinely used) ( Guyon *et al.,* 2009). IBFAT also had a low inter-rater reliability. Furthermore, most studies were low quality (e.g. small sample size and observational designs) and were also conducted exclusively in high income settings ( Furman & Minich, 2006; Matthews, 1988; Matthews, 1991b; Riordan & Koehn, 1997; Schlomer *et al.,* 1999). 

Nine tools were tested for test-retest and inter-rater reliability in eight studies - one study compared three tools. Two tools performed well: the Integrated Management of Childhood Illness (IMCI) showed good sensitivity and high specificity in highlighting breastfeeding problems judged against clinician assessments ( Darmstadt *et al.,* 2009); the Mother Infant Breastfeeding Progress Tool (MIBPT) showed high inter-rater agreement ( Johnson *et al.,* 2007). There were mixed findings for the remaining tools. Details of these studies are in Table 4.

**Table 4.** Studies assessing breastfeeding assessment tool reliability.

Assessment Tool	Author(s) & date	Country & setting	Sample	Study design	Outcomes	Findings	Remarks
IMCI algorithm	( Darmstadt *et al.,* 2009)	Bangladesh, community	N=395 neonates aged 0-8 days	Observational	Validity of community health worker identified symptoms and signs of illness/ feeding problems (against clinician ‘gold standard’ opinion)	Health worker classifications had 73% sensitivity, 98% specificity, 57% positive and 99% negative predictive value. Identified feeding problems: ‘not sucking at all’, ‘not attached at all’, ‘not well attached’ were all confirmed by physician questioning of mother	There is no gold standard for breastfeeding assessment and no evidence that physician questioning is superior to IMCI
Infant Breastfeeding Assessment Tool (IBFAT)	( Riordan & Koehn, 1997)	USA, hospital and community	N=11 breastfeeding women and their newborns children	Observational	Inter-rater (n=3) and test-retest reliability of 3 breastfeeding assessment tools from n=12 randomly selected videoed feeds	Spearman rank order coefficients of inter-rater correlations ranged from .27 to .69 for IBFAT. Test-retest correlation=r0.88.	Small number of observations are unlikely to be representative
LATCH	( Adams & Hewell, 1997)	USA, hospital	n=35 first time breastfeeding mothers	Observational	Inter-rater reliability of lactation consultant scores and mothers’ LATCH scores	85-100% lactation consultant agreement. Correlation with maternal reports=very low-moderate	Mothers may focus on somatic experience
LATCH	( Riordan & Koehn, 1997)	USA, hospital and community	N=11 breastfeeding women and their newborns children	Observational	Inter-rater (n=3) and test-retest reliability of 3 breastfeeding assessment tools from n=12 randomly selected videoed feeds	Spearman rank order coefficients of inter-rater correlations ranged from .11 to .46. The reported test- retest correlation was .88.	Small number of observations unlikely to be representative
Mother-Baby Assessment (MBA)	( Riordan & Koehn, 1997)	USA, hospital and community	N=11 breastfeeding women and their newborns	Observational	Inter-rater (n=3) and test-retest reliability of 3 breastfeeding assessment tools from n=12 randomly selected videoed feeds	Spearman rank order coefficients of inter-rater correlations ranged from r=0.33 to 0.66; test-retest correlation r=0.88.	Small number of observations unlikely to be representative
Mother-infant breastfeeding progress tool (MIBPT)	( Johnson *et al.,* 2007)	USA, Hospital	N=62 healthy mother- baby pairs; 35-42 weeks gestational age. Infants 2 hours-5 days old	Observational	Inter-rater agreement of tool scores	Inter-rater agreement: 79-95%.	No maternal or child outcome included
NOMAS	( Palmer *et al.,* 1993)	USA, Hospital	N=35 infants, 35-49 weeks post menstrual age, ≥1900g	Observational	Percentage agreement of 3 coders	Inter-rater reliability: 80% agreement.	
NOMAS	( Da Costa *et al.,* 2008)	Holland, setting not stated	N=75 healthy & very low birth weight infants; 26-36 post menstrual age	Observational	Inter-rater agreement	Test-retest of NOMAS with 4 raters = moderate to near perfect (r=0.33-0.94)	Tool could incorporate new knowledge of infant suck/swallow
NOMAS	( Howe *et al.,* 2007)	USA, medical centre	N=147 preterm, but healthy infants, 32-26 weeks post menstrual age	Observational	Infant feeding performance: transitional rate & volume of milk consumed from bottle.	Acceptable reliability of normal & disorganized categories. All categories moderately correlated with transitional milk rate.	
PIBBS	( Nyqvist *et al.,* 1999)	Sweden, hospital	N=24 full/ preterm infants in neonatal intensive care, transitional/ maternity units.	Observational	Inter-rater reliability of observers, & observers/ mothers.	Good inter-rater reliability for observers (r=0.64-1.00), but poor for observers and mothers (r=0.27-0.86). Poor items revised.	Unclear analysis testing tool detection of gestational age/maturity of breastfeeding
Infant Breastfeeding Assessment Tool (IBAT) and LACTH and modified Via Christi (mVC) and Riordan's tool (RT)	( Chapman *et al.,* 2016)	USA	N=45 participants overweight and obese women, multiparas, Latinas	Observational	Inter-rater reliability of 4 lactation assessment tools applied to overweight and obese women. Swallowing evaluation was unreliable especially during the first week of life.	Inter-rater reliability was evaluated with 3 methods analisys of variance (ANOVAs) - average measures intraclass correlation coefficients (ICCs) – percentage absolute agreement between raters.	
Bristol Breastfeeding Assessment Tool (BBAT)	( Ingram *et al.,* 2015)	UK	N=34 dyads under 2 weeks old infants	observation and qualitative	inter-rater reliability with Cronbach's alpha	high correlation in consistency	
Bristol Breastfeeding Assessment Tool (BBAT)	( Dolgun *et al.,* 2018)	Turky, Hospital	N=127 mothers of 0-6months old baby	observational of 2 paediatric nurses	inter-rater agreement with Kappa analysis. Consistency over time analysis and item analysis.	Strongly significant agreement between the two raters in terms of "positioning", "lacting" and "sucking" domains and significant agreement in terms of "swallowing" domain	
Early Feeding Skills Assessment Tools (EFSAT)	( Thoyre *et al.,* 2018)	USA, Hospital	N=8 cases of 2 months old baby	Observational	Inter-rater reliability with Cronbach’s alpha	Cronbach’s alpha was 0.81 indicating acceptable internal consistency on EFS total scale.	

### Ability of tools to correct breastfeeding technique or improve breastfeeding experience

Few studies tested the use of tools to correct breastfeeding technique or to improve breastfeeding experience. These are shown in Table 5.

**Table 5.** Studies assessing the ability of breastfeeding assessment tools to improve breastfeeding technique or experience.

Assessment Tool	Author(s) & date	Country & setting	Sample	Study design	Outcomes	Co-variates	Findings	Remarks
IMNCI guidance	( Thakre *et al.,* 2012)	India, Hospital	N=104 babies	Observational	Breastfeeding position and attachment	Not clear	Significant improvements to breastfeeding positioning and attachment observed after IMNCI assessment and guidance	
IMNCI guidance	( Dongre *et al.,* 2010)	India, Community	N=99 mothers and children <6 months	Observational	Child feeding problems	None	Significantly more women had an observable positioning and/or attachment difficulty than other feeding problems	
Infant Breastfeeding Assessment Tool (IBFAT)	( Matthews, 1991a)	Canada, hospital	N=56 healthy breastfeeding mothers and their newborns	Observational	Maternal satisfaction with breastfeeding	Considered multi and primiparous separately	Higher ‘effective feeding’ scores linked to greater maternal satisfaction. Primiparous rated infants lower and were more dissatisfied than multiparous mothers.	Nurses observed 77/812 feeds to assess reliability: <10% cases were significantly different
Infant Breastfeeding Assessment Tool (IBFAT)	( Furman & Minichm, 2006)	USA, hospital	N=34 mothers of very low birth weight infants; 35 weeks gestational age	Observational	Milk-intake (test-weighing)	None	IBFAT scores positively correlated with feeding observations and milk intake; sucking score correlated with percentage time suckling	IBFAT does not discriminate adequate and inadequate milk intake.

## Discussion

Our review identified a number of breastfeeding assessment tools which could be used in the management of our target group of at-risk and malnourished infants aged under 6 months. Though none of the tools were developed for or tested on this group directly, characterising them and understanding the underlying evidence-base allows for better informed decisions about which might be the most helpful for future programme use.

Regarding the coverage of breastfeeding domains, only one tool (BEET) achieves full coverage of all the key assessment domains, but there were no validation study at our knowledge. The tools that achieve the widest coverage (IFE Module 2, BEET, and WHO/UNICEF B-R-E-A-S-T-Feed Observation Form and UNICEF/WHO Breastfeed Observation Aid) are generally those which have been developed with resource-poor low and middle income countries in mind. Although these tools are based on extensive clinical and field experience, they suffer from lack of validation research and miss some important domains (e.g. WHO/UNICEF B-R-E-A-S-T-Feed Observation Form misses health of the baby, IFE Module 2 misses positioning). These shortfalls could be addressed with minor modifications in the short term and with appropriately designed studies soon after to help determine which domains are the most important and relevant to patient care. Only 11 tools assess mothers’ own behaviour towards the baby: this is telling about her psychosocial status and can inform management. It is important to consider and account for such gaps since an infant may be effectively breastfed but at risk and malnourished for another reason, e.g. related to child health status or maternal factors. The mother-infant dyad is at the heart of approaches to treat malnutrition, but wider family and community relationship are also important but cannot be treated extensively in this review ( ENN/LSHTM, 2021b).

A challenge validating breastfeeding assessment tools is the lack of a ‘gold standard’ treatment option for at-risk and malnourished infants less than 6 months. This makes validation studies a challenge methodologically since it is difficult to separate out the performance of an assessment tool from the effectiveness of the subsequent management strategy in averting adverse nutrition/morbidity outcomes. It is likely that different tools and different levels of management will be appropriate to different settings, e.g.

- **In primary healthcare / community settings**: simple and rapid breastfeeding assessment tools, associated with easy-to-deliver interventions and to prompt referral for more specialised support. For use by community healthcare workers who may have limited training and experience.
- **In secondary healthcare / outpatient clinic settings**: more detailed tools could be appropriate but would need more training and staff with more background skills, expertise and time to deliver.
- **In tertiary-level inpatient settings**: more complex assessments would be appropriate to identify more complex problems. These could be delivered by more highly trained healthcare staff such as nurses and doctors.

No single tool meets all these needs. Which tool is more appropriate to a given setting and individual mother-infant situation is itself an important question that warrants further testing and exploration.

For immediate use, whilst refining current tools and developing new future ones, the WHO/UNICEF B-R-E-A-S-T-Feed Observation Form, the aids in Module 2 on IFE and UNICEF/WHO Breastfeed Observation Aid, offer the most promise for programmes targeting at-risk and malnourished infants aged under 6 months.

In future research testing current and new tools, there is a need to agree on the most appropriate outcomes for validation studies targeting at-risk and malnourished infants under 6 months. The fact that so many tools exist, and that they cover such a wide range of feeding outcomes and domains arguably reflects uncertainly and lack of consensus about how best to assess the effectiveness of breastfeeding. For example, must there always be sufficient infant weight gain associated with other measures of effective feeding? Most current evidence comes from high-income countries and hospital settings. For use in tackling the significant global burden of malnutrition in infants aged less than 6 months, this is a problem. More tools for low income countries and for community settings are urgently needed ( Moran *et al.,* 2000; Mulder, 2006; Riordan, 1998; Riordan & Koehn, 1997).

Another key finding of our review was the variable - and overall low - quality of evidence underpinning existing breastfeeding assessment tools. Often the evidence-base for a particular tool is unclear, particularly their effectiveness in identifying specific breastfeeding problems and facilitating a resolution. Prospective and ideally randomised studies testing tools’ ability to do this are important in the future ( Da Costa *et al.,* 2008). Simple checklists have been shown to be powerful if used consistently in clinical settings ( Haynes *et al.,* 2009; Pronovost *et al.,* 2006). There is therefore an argument to develop checklist-based tools that can be incorporated into routine breastfeeding assessment, to maximize the chances of resolving breastfeeding problems. These should also be able to discriminate between different types of breastfeeding problems and lead clearly to specific interventions. 

We found that tools varied in their level of complexity, and their scoring systems. This may make individual tools relevant only for specific contexts. For example, three tools involve two stages: IFE Module 2 includes a simple rapid assessment, followed by a full assessment ( ENN *et al.,* 2007); the BFHI guidelines may include initial use of the breastfeeding assessment form, leading on to the UNICEF/WHO breastfeed observation aid if necessary ( UNICEF, 2010; WHO/UNICEF, 2009a); the IMCI algorithm includes both a brief history taking and observations of the breastfeed ( Mannan *et al.,* 2008). This is potentially a good thing. Rather than one tool trying to do everything, different tools for different levels of assessment could be helpful: e.g. a quick, basic tool for use in the community to identify and correct ‘simple problems and identify referral need, complemented by a more detailed tool if problems are suspected or identified; another more detailed one for clinic/hospital use assessing more serious and complex problems flagged by the first tools. Tool developers need to consider what the key contact points with infants are, and the associated opportunities and capacities with these contact points. Coupled with this must be the capacity to respond to any problems identified. To address breastfeeding in high mortality/morbidity settings, tools need to consider not just physiological issues and techniques around breastfeeding, but also the wider social and psychological factors, which may be contributing to or perpetuating a problem ( Galipeau *et al.,* 2017).

### Which tools for resource-poor, high-undernutrition settings

From this review, Baby Friendly Hospital tools, the Module 2 IFE and WHO/UNICEF B-R-E-A-S-T-Feed Observation Form, have emerged as potentially useful for use in humanitarian settings with at-risk and malnourished infants under 6 months. They require a short training and they are easy-to-use. Baby Friendly Hospital tools and the Module 2 IFE could benefit from adaptation by adding the missing components that we would be considered useful for humanitarian contexts. While BFHI has become a ‘gold standard’ for maternity care in hospital setting, the effectiveness of the training course has been assessed but the evaluation of the breastfeeding assessment form requires more studies. Equally, these tools could be combined (e.g. by adding questions from one tool to another) in a way that might improve the quality of breastfeeding assessment, and that would take into account the specific needs and limitations of contexts with a high burden of undernutrition. It will be important to ascertain the feasibility of community health workers using these tools.

Based on coverage of domains, appropriateness to target population and setting, and underlying evidence, WHO/UNICEF B-R-E-A-S-T-Feed Observation Form appears to be the most suitable for assessing at risk and malnourished infants aged under 6 months. In two Danish RCTs, health visitors were trained to conduct home visits incorporating breastfeeding assessment and classification of technique problems ( Kronborg & Vaeth, 2009; Kronborg *et al.,* 2007). One study found a 14% lower breastfeeding cessation rate amongst intervention participants, and greater confidence of mothers that their breast milk was sufficient. However, the other found no difference in exclusive breastfeeding rate or a reduction in breastfeeding problems - this may be due to a single corrective intervention being insufficient to resolve breastfeeding problems. The authors argued for on-going breastfeeding support to ensure breastfeeding problems are truly resolved. This idea is corroborated by a third Brazilian hospital-based RCT with a low socioeconomic population, which found no impact of a single breastfeeding assessment and correction on exclusive breastfeeding rates, breastfeeding technique or breastfeeding problems 30 days post-partum ( De Oliveira *et al.,* 2006). A further RCT in Brazil also used the WHO/UNICEF B-R-E-A-S-T-Feed Observation Form but included a greater number of home visits (n=6). This observed a 39% increase in any breastfeeding, and a significant increase in exclusive breastfeeding. One limitation of this study is that it is difficult to unpick the effect of the breastfeeding observation and corrective advice from the other interventions during the home visit ( Leite *et al.,* 2005). This underlines the importance of not just having a good tool, but using it to maximum effect i.e. not just conducting a single assessment and correction, but providing on-going support through community outreach ( Imdad *et al.,* 2011). What is most encouraging about the WHO/UNICEF B-R-E-A-S-T-Feed Observation Form is its apparent usability in routine clinical settings, with relatively short training if conducted for the use of the test only. As the tool is part of a broader training on breastfeeding counselling, it is recommended to explore the whole manual, but it is also possible to adapt to the situation’s needs. It would still be valuable to do further validation of this tool and possibly extend the tool components to include aspects of the baby’s health, as identified in the section on coverage of breastfeeding domains.

## Ways forward

As well as standard validation studies, new tools or those initially developed in/adapted from resource-rich settings should be assessed for cultural relevance and sensitivity before they are considered for use in resource-poor developing country/humanitarian settings. This formative work should ideally precede detailed validation or intervention studies. Validity is likely to vary according to target patient group and studies should therefore be sufficiently powered to explore subgroups. Tools that are designed to assess breastfeeding in healthy, well-nourished infants are not necessarily as good or adequate for assessing sick or undernourished ones. As none of those tools presented above were developed and tested in malnourished children and since these infants are at particularly high risk of morbidity and mortality, specific tools should consider the needs of infants aged less than 6 months with malnutrition – the group who inspired this review in the first place. Since there are many factors potentially underlying or contributing to malnutrition, we believe that tools for this group should be part of a wider assessment of the mother-infant dyad and take an appropriately broad perspective by considering other factors known to impact on infant nutrition e.g. maternal mental health, maternal illness, and maternal malnutrition.

## Limitations

We acknowledge the limitation of our review. Firstly, it was restricted to articles written in English; there may be useful breastfeeding assessment tools published in other languages that were not captured.

Secondly, it is possible that we missed some studies, e.g. those using a broader approach to improving infant feeding may not have explicitly mentioned breastfeeding assessment tools as part of their intervention protocol; those which were using a tool in a programme but were not in the title or abstract clearly evaluating/testing the tool itself; those that may have had relevant content (e.g. maternal psychosocial status) but did not meet the inclusion criteria of one clinically relevant maternal or child outcome.

Third, we did not explicitly grade the quality of individual studies – this was felt not to add significant extra value to our review since observational studies, which comprised great majority of papers identified, are by definition low quality compared to intervention/RCT type designs. Quality grading would not have helped differentiate between more/less valuable tools, since the quality of evidence underpinning them all was generally low.

Finally, we found few tools explicitly targeted to our setting and main patient group of interest. This is not ideal since it means applicability had to be extrapolated based on our judgement rather than on hard data.

Despite these limitations, we do not believe that the overall direction or message arising from our findings are affected.

## Conclusion

In this review of breastfeeding assessment tools for resource poor settings and targeting the assessment of malnourished infants less than 6 months, we have identified many possible but few stand-out ‘gold standard’ options. This represents an important evidence gap and highlights an urgent need for future research. The many different tools that we did find arguably show that one tool alone is unlikely to be suitable or even desirable. Tools must strike the right balance between simplicity, feasibility of use and minimal training requirements without losing the depth of information required to help healthcare workers and the women they are working with address breastfeeding difficulties. Thus, different tools for different levels of the health care system are needed: simple, quick-to-use tools for initial triage and problem identification in the community; more sophisticated tools for use in secondary and tertiary care settings where initial attempts at support have failed. Supplementary items such as pictures of good latch, and materials to help mothers and health workers understand the nature of breastfeeding problems (e.g. ‘take action cards’ ( Dongre *et al.,* 2010)), may be helpful. For any tool at any level, it is important that it leads to clear corrective actions. A “diagnosis” or “problem label” by itself is not always useful. Hence, future tools might give appropriate weight to problems, which can most readily be solved, or those which have the biggest short and long term impact. Research on breastfeeding assessment tools needs to consider such impacts – again, good test inter- and intra-observer validity is necessary but not alone sufficient to make a ‘good’ tool. It must help improve key outcomes like breastfeeding status and infant growth. Robustly designed studies in the contexts in which they will be used are essential.

Finally, we note that time will be needed to develop and test better future breastfeeding assessment tools. Yet support for women and their infants is urgently needed now. Not having an ideal tool is not a reason to defer breastfeeding assessment of at risk and malnourished infants under 6 months. There are great opportunities at present to collect and report good quality operational data using tools that are currently available. Expanding the current literature on breastfeeding assessment will be of great benefit to future tool developers. More importantly, focus on this area will also raise the profile of and directly benefit breastfeeding as a key child nutrition, health and survival intervention.

## Data availability

### Underlying data

All data underlying the results are available as part of the article and no additional source data are required.

### Extended data

LSHTM Data Compass: Breastfeeding assessment tools for at-risk and malnourished infants aged under 6 months old: a systematic review, https://doi.org/10.17037/DATA.00001881 ( Kerac *et al.,* 2020).

This project contains the following extended data:

- Tools excluded from the second stage of the literature search
- Full search strategy

### Reporting guidelines

LSHTM Data Compass: PRISMA checklist for ‘Breastfeeding assessment tools for at-risk and malnourished infants aged under 6 months old: a systematic review’, https://doi.org/10.17037/DATA.00001881 ( Kerac *et al.,* 2020).

Data are available under the terms of the Creative Commons Attribution-NonCommercial 2.0 UK license (CC BY-NC 2.0 UK).
