# Supplementary material for: Breastfeeding assessment tools for at-risk and malnourished infants aged under 6 months old: a systematic review
Source: F1000Res. 2021 Oct 22;9:1310. Originally published 2020 Nov 10. [Version 2] doi: 10.12688/f1000research.24516.2 (PMC7898355; doi:10.12688/f1000research.24516.2)
Supplement: Peer review history

# Peer review: Reviewer response for version 1

**DOI:** 10.5256/f1000research.27043.r77936

**Published:** 2021-02-19

**Authors:** Nurul Husna Mohd Shukri

**Affiliations:** 1 Department of Nutrition and Dietetics, Faculty of Medicine and Health Sciences, Universiti Putra Malaysia, Seri kembangan, Malaysia

The review compares different breastfeeding assessment tools to identify suitable tools to be used among at-risk and malnourished infants. The review also evaluates the breastfeeding assessment tools by comprehensively discussing both advantages and limitation of each tool and its reliability, as well as its validity in assessing breastfeeding outcomes. This review emphasizes the need for future tools to suit different breastfeeding management levels and settings, from primary to tertiary settings.

 The authors clearly outline the study limitations and provide important suggestions in developing comprehensive and target-setting specifics of future breastfeeding assessment tools. All tables are clear and well-organised.

 Overall, this review critically analyses various important criteria of breastfeeding assessment tools in different settings, addressed important key gaps, and provides suggestions in establishing a better version of the tools in the future. All of these would provide a significant value to the literature.

 Nevertheless, there are a few minor suggestions to consider:

1. It is suggested to describe the key term used or provide a table showing all Medical Subject Heading (MeSH) and keyword used for literature search.
2. Clarify or describe the term at-risk and malnourished infants.
3. Differentiate or explain the difference/similarity between breastfeeding outcomes and performance.
4. It is suggested for authors to proofread the paper to improve the overall writing.

Are the rationale for, and objectives of, the Systematic Review clearly stated?

Yes

Is the statistical analysis and its interpretation appropriate?

Not applicable

Are sufficient details of the methods and analysis provided to allow replication by others?

Yes

Are the conclusions drawn adequately supported by the results presented in the review?

Yes

Reviewer Expertise:

Mother-infant signalling, relaxation therapy during breastfeeding, breast milk hormones

I confirm that I have read this submission and believe that I have an appropriate level of expertise to confirm that it is of an acceptable scientific standard.

# Response

**Published:** 2021-05-25

**Authors:** Concetta Brugaletta

**Affiliations:** University College London Hospitals NHS Trust, UK

Thank you very much for taking the time to read the review and for all your comments, which we find it constructive and  useful to clarify some concepts as below. 

 1. Regarding your first comment on describe the key term used or provide a table showing all Medical Subject Heading (MeSH) and keyword used for literature search; The search-strategy is deposited at LSHTM Data Compass as part of the Extended Data. Extended data contains tools excluded from the second stage of the search, full search strategy and PRISMA checklist. This was pointed out in the reference *“ Kerac M, Brugaletta C , Le Roch K: Breastfeeding assessment tools for at-risk and malnourished infants aged under 6 months old: a systematic review. [Data Collection]. London School of Hygiene & Tropical Medicine, London, United Kingdom.2020.* *http://www.doi.org/10.17037/DATA.00001881* *”* We have added the link in the methods to make it easier to access.

**2. **Regarding your second comment to clarify or describe the term at-risk and malnourished infants; “ *A group with higher risk of mortality and morbidity are the small and nutritionally at risk infants under six months of age compared to the infant that achieve optimal growth. At a population level, small and nutritionally at-risk children are those identified as wasted, stunted and underweight and a combination of these (ENN/LSHTM, 2021)* *”*. We added a reference in the text of the larger project that clarify this concept: https://www.ennonline.net/ourwork/research/mami

**3. **Regarding your third comment on differentiate or explain the difference/similarity between breastfeeding outcomes and performance; We decided to modify the phrasing and replaced by: ‘ *how to observe and/or assess the breastfeeding performance with outcomes*’.

**4. **Regarding your suggestion for authors to proofread the paper to improve the overall writing; We have now proofread and think this looks fine in this last version. the major changes are: 

 Table 2: corrected ‘behaviour’

 Table 3: corrected ‘postnatal’; ‘paediatric’

 Table 4: corrected ‘analysis’

 Text and table 1: corrected ‘lactating’ ( https://www.macmillandictionary.com/dictionary/british/lactate

 Text corrected ‘paediatric’

# Peer review: Reviewer response for version 1

**DOI:** 10.5256/f1000research.27043.r78113

**Published:** 2021-02-18

**Authors:** Sandra Fucile

**Affiliations:** 1 School of Rehabilitation Therapy, Queen's University, Kingston, ON, Canada

Brugaletta *et al.'s*, review of breastfeeding assessment tools for assessing at-risk and malnourished infants in resource-poor settings provides a comprehensive literature search of available tools for this highly vulnerable population. The authors reveal there is no ‘gold standard’ tool available for at-risk and malnourished infants in resource-poor settings. However, they highlight three ready available tools, the Breastfeeding, Evaluation and Education Tool, UNICEF Baby-Friendly Hospital Initiative tools and CARE training package, that can be used with this population and emphasize the need for refining or developing new breastfeeding tools to meet the needs of infants in resource-poor settings.

 Overall, the authors provide a very thorough introduction with a clear rationale for undertaking this study. The authors perform a systematic in-depth literature search, which included seven online database resources. The authors identified 29 breastfeeding assessment tools and 45 studies related to the tools' psychometric properties. They found that the evidence and psychometric properties of the tools was low quality and mainly from high-income countries. The strengths and weakness of these 29 breastfeeding tools were described in terms of the tool content of breastfeeding domains, predictive validity, reliability, and evidence underlying the content each tool. The tables provided clearly synthesize and integrate the strengths and weakness of each tool. In the discussion, the authors address the limitations of the study and bring to light the drawbacks of current available tools in achieve the defined outcome in this study.

 The findings from this review are clinically significant and I have minor suggestions:

- I encourage the authors to thoroughly reread the manuscript to ensure there are no grammatical and editorial errors.
- Figure 1 appears to be adopted from the PRISMA framework, ensure that this is referenced.
- Use of terminology, the authors refer to Table 3 as validation studies, I recommend rewording to use predictive validity studies.
- I recommend including specific definitions for high vs low income countries, define at-risk infants, define malnourished infants either in the introduction or methods section.
- The authors note 29 tools were identified, 22 were developed in high income countries, 4 low income countries. Three tools are missing such designations.

Are the rationale for, and objectives of, the Systematic Review clearly stated?

Yes

Is the statistical analysis and its interpretation appropriate?

Yes

Are sufficient details of the methods and analysis provided to allow replication by others?

Yes

Are the conclusions drawn adequately supported by the results presented in the review?

Yes

Reviewer Expertise:

Oral feeding in critically ill infants.

I confirm that I have read this submission and believe that I have an appropriate level of expertise to confirm that it is of an acceptable scientific standard.

# Response

**Published:** 2021-05-25

**Authors:** Concetta Brugaletta

**Affiliations:** University College London Hospitals NHS Trust, UK

Thank you for taking the time to read the review and write your suggestion.

 Regarding your advice to proofread the manuscript to ensure there are no grammatical and editorial errors; We have proofread and we think this looks fine in the new version of the manuscript.

 Regarding your advice to ensure the reference on Figure 1; we added the reference at the base of the figure in the new version of the manuscript. 

 Regarding your suggestion on the use of a different terminology for Table 3; We hope it is okay to stick with validation studies as it is commonly used in the literature. For reference, see: https://www.equator-network.org/reporting-guidelines-keyword/validation-studies/

 Regarding your recommendation to include a  specific definitions for high vs low income countries, define at-risk infants, define malnourished infants either in the introduction or methods section; We added the following references that will help clarifying the definition and the concept as follow:

 - High and low income countries: as classified by The World Bank

*Fantom, Neil; Serajuddin, Umar. 2016. The World Bank's Classification of Countries by Income. Policy Research Working Paper;No. 7528. World Bank, Washington, DC. © World Bank. https://openknowledge.worldbank.org/handle/10986/23628 License: CC BY 3.0 IGO.*

 - At-risk infants: https://www.ennonline.net/ourwork/research/mami

 Regarding your comment "29 tools were identified, 22 were developed in high-income countries, 4 low income countries. Three tools are missing such designations"; The tools that do not give any information about country of origin (described in the table 1 as not specified) are:

 - Baby-friendly Hospital Initiative (BFIH)

 Worldwide (When the Baby-friendly Hospital Initiative was conceived in the early 1990s in

 response to the 1990 Innocenti Declaration on the Protection, Promotion and Support of

 Breastfeeding call for action, there were very few countries that had dedicated

 Authorities or Committees to oversee and regulate infant feeding standards.)

 - Essential Nutrition Action Message (Guyon & Quinn 2011)****

 Low and middle-income countries

 - Infant Feeding in Emergency (IFE) (ENN 2007)

 Low and middle-income countries

 - Neonatal Eating Assessment tool (NeoEAT)****

 This is a USA based organisation: AWHONN, the Association of Women's Health, Obstetric and Neonatal Nurses. https://www.awhonn.org/

 We amended the information above in table n1 of the new version of the manuscript. In the text of the new version of the manuscript we clarify the follow information: *“Of the 29 tools identified: 22 (76%) were developed in high-income countries and used in 31 studies carried out in high-income countries, six (21%) tools were developed in low and middle-income countries and one (3%) was developed worldwide”.*

# Peer review: Reviewer response for version 1

**DOI:** 10.5256/f1000research.27043.r74628

**Published:** 2020-12-01

**Authors:** Kerstin E. Hanson, Jessamyn Ressler-Maerlender

**Affiliations:** 1 Independent researcher, Paris, France; 2 Independent researcher, Ann Arbor, MI, USA

This review by Brugaletta *et al. *addresses the important gap in tools and evidence for effective case management of at-risk and malnourished infants aged under 6 months old in low-resource and humanitarian settings. The authors focus specifically on the availability and quality of breastfeeding assessment tools for use in this population. They start with an excellent introduction highlighting the importance of this theme – describing the essential role of breastfeeding in protecting the health and lives of children and the subsequent place it has in global priority interventions, and the challenges that remain in properly addressing persistently suboptimal breastfeeding practices.

 Results of the review are presented in clear tables, summarizing important features of the various tools identified. The authors provide a clear breakdown of these features into the following categories: context, coverage of breastfeeding domains, ability to predict breastfeeding outcomes, evidence underpinning the tools, and ability to correct breastfeeding technique or improve breastfeeding experience. The analysis also addresses not only technical or academic features of the tools, but also “real world” implementation issues, and ability to bring about the desired outcomes - improved breastfeeding.

 The discussion highlights strengths and gaps of individual tools, as well as the overall “collection” of tools identified. The authors are clear to state that none of the tools were directly developed for or tested on at-risk and malnourished infants aged under 6 months, nor do any of the tools fully meet the various needs in terms of categories outlined above. The authors do nevertheless identify three tools that could be used “for immediate use, whilst refining current tools and developing new future ones”.

 We appreciate the overall approach to this review – identifying and analyzing current tools, recognizing that we do not currently have an ideal tool, explaining the key gaps and ways forward, and importantly – providing temporary best options. Minor suggestions to consider in subsequent versions:

1. In the abstract the authors list the following as part of their search: the World Health Organization (WHO), United Nations International Children’s Emergency Fund (UNICEF), CAse REport guidelines, Emergency Nutrition Network, and Field Exchange websites as parts of the search. These are not mentioned in the database and search terms of the methods section. They are perhaps listed in one of the references, but it might be helpful to include them in this later section of the manuscript as well.
2. In figure 1 it is unclear where the “Handsearch Papers” fit. This part of the search could be expanded upon in the methods section.
3. Under context the authors state: “Of the 29 tools identified: 22 (76%) were developed in high-income countries and used in 31 studies carried out in high-income countries and four (14%) tools were developed in low and middle-income countries.” What about the 3 tools, not included in the 22 developed in high-income countries and the 4 developed in low- and middle-income countries?
4. The introduction does a nice job addressing the particular challenges associated with “managing very smalll infants, those with growth failure and other high-risk characteristics”. It also highlights the complex spectrum of breastfeeding problems including potential underlying causes and contributory factors, including but not limited to maternal wellbeing and social support. Although these essential topics are touched upon very briefly in the results and discussion sections, and in a bit more detail in the ways forward section, the review could benefit with expansion of these critical topics.
5. In the discussion section the authors note that only one tool, BEET, achieves full coverage of all the key assessment domains. Yet, this tool is not included amongst those listed as potentially useful for immediate use; it could be useful to note why this is the case.
6. In the initial paragraphs of the discussion, the authors suggest: “For immediate use, whilst refining current tools and developing new future ones, the WHO/UNICEF B-R-E-A-S-T-Feed Observation Form, the aids in Module 2 on IFE and UNICEF/WHO Breastfeed Observation Aid, offer the most promise for programmes targeting at-risk and malnourished infants aged under 6 months”. Later, under the heading *Which tools for resource-poor, high-undernutrition settings*, they suggest: “From this review, Baby Friendly Hospital tools, the Module 2 IFE and WHO/UNICEF B-R-E-A-S-T-Feed Observation Form, have emerged as potentially useful for use in humanitarian settings with at-risk and malnourished infants under 6 months.“ Referring to the tools listed in the tables, we imagine that the Baby Friendly Hospital tools and UNICEF/WHO Breast Observation Aid noted above are referring to the same tool. If so, the same naming convention should be used in both instances for clarity.
7. There are a number of minor typos and grammatical errors throughout the paper and tables that should be corrected.

Are the rationale for, and objectives of, the Systematic Review clearly stated?

Yes

Is the statistical analysis and its interpretation appropriate?

Not applicable

Are sufficient details of the methods and analysis provided to allow replication by others?

Partly

Are the conclusions drawn adequately supported by the results presented in the review?

Yes

Reviewer Expertise:

Pediatric and nutrition programming and case management in low-resource and humanitarian settings.

We confirm that we have read this submission and believe that we have an appropriate level of expertise to confirm that it is of an acceptable scientific standard.

# Response

**Published:** 2021-05-25

**Authors:** Concetta Brugaletta

**Affiliations:** University College London Hospitals NHS Trust, UK

Thank you very much for taking the time to read the manuscript and share your comments and constructive advice.

 1. Regarding your first comment where you point out that we mentioned the World Health Organization (WHO), United Nations International Children’s Emergency Fund (UNICEF), CAse REport guidelines, Emergency Nutrition Network, and Field Exchange websites as parts of our search but we didn't mentioned these in the database and search terms; we have now clarified this in the methods search under the databases and search terms paragraph as follow: “We also included hand search papers form grey literature, WHO and ENN websites”.

 2. Regarding your second comment for figure n1 where was unclear where the “Hand search Papers” fit; we amended figure n1 in the new version of the manuscript. We also add a reference *Preferred Reporting items for Systematic Reviews and Meta-Analyses (PRISMA) diagram of literature search results. Diagram retrieved from:* *http://prisma-statement.org/PRISMAStatement/FlowDiagram.aspx*

 3. Regarding your request of clarification for the paragraph “Of the 29 tools identified: 22 (76%) were developed in high-income countries and used in 31 studies carried out in high-income countries and four (14%) tools were developed in low and middle-income countries." 

 Three tools are missing such designations"; The tools that do not give any information about country of origin (described in the table 1 as not specified) are:

 - Baby-friendly Hospital Initiative (BFIH)

 Worldwide (When the Baby-friendly Hospital Initiative was conceived in the early 1990s in

 response to the 1990 Innocenti Declaration on the Protection, Promotion and Support of

 Breastfeeding call for action, there were very few countries that had dedicated

 Authorities or Committees to oversee and regulate infant feeding standards.)

 - Essential Nutrition Action Message (Guyon & Quinn 2011)

 Low and middle-income countries

 - Infant Feeding in Emergency (IFE) (ENN 2007)

 Low and middle-income countries

 - Neonatal Eating Assessment tool (NeoEAT)

 This is a USA based organisation: AWHONN, the Association of Women's Health, Obstetric and Neonatal Nurses. https://www.awhonn.org/

 We amended the information above in table n1 of the new version of the manuscript. In the text of the new version of the manuscript we clarify the follow information: *“Of the 29 tools identified: 22 (76%) were developed in high-income countries and used in 31 studies carried out in high-income countries, six (21%) tools were developed in low and middle-income countries and one (3%) was developed worldwide”.*

 4. Regarding your comment on considering complex spectrum of breastfeeding problems including potential underlying causes and contributory factors, including but not limited to maternal wellbeing and social support; It is a great suggestion; the number of words required does not allow us to expand on this very interesting and crucial topic. We have however added a reference of the MAMI website, which is regularly being updated and a note saying *“The mother-infant dyad is at the heart of approaches to treat malnutrition, but wider family and community relationship are also important but cannot be treated extensively in this review”. *(ENN/LSHTM, 2021) https://www.ennonline.net/ourwork/research/mami

https://www.ennonline.net/mami/practice

 5. Regarding your comment on the discussion section where only one tool, BEET, achieves full coverage of all the key assessment domains. Yet, this tool is not included amongst those listed as potentially useful for immediate use; Thank you for your comments. The justification is in the paragraph on evidence underpinning tools: ‘ *The extent of tool testing varied substantially; 8 tools had no validation studies: Infant Feeding in Emergencies (IFE) Module 2* *(* *ENN et al., 2007* *)* *, Breastfeeding Evaluation and Education Tool* *(* *Tobin, 1996* *)*’. This is why we do not include for immediate use.  We have added a note in the text “only one tool (BEET) achieves full coverage of all the key assessment domains, but there was no validation study at our knowledge”.

 6. Regarding your comment on request of clarification if the Baby Friendly Hospital tools and UNICEF/WHO Breast Observation Aid are referring to the same tool. We would like to explain their difference and the rational for our subtle considerations: 

 - The Baby Friendly Hospital tools: is a checklist (12 to 14 items) designed with the aim to identify area of problem and give advice. These tools take in consideration health professional background and day of life of the baby and one can also be self-administered. This means there are 4 slightly different tools available: for mother and midwife, for mother and health visitor, for neonatal and for mother alone. The domain covered are:  baby’s and mother behaviour, positioning, lactating, effective feeding, breast health, baby health, mothers view (in addition these tools look at urine and stools, formula). ( https://www.unicef.org.uk/babyfriendly/baby-friendly-resources/implementing-standards-resources/breastfeeding-assessment-tools/).

 - The UNICEF/WHO Breastfeeding Observation Aid is a checklist of identify dichotomous items ( 42 items/5 scales) list signs that represent that BF is going well versus possible difficulties. It cover similar breastfeeding domains of the BFH tools but is a more simple checklist and doesn’t offer possible solutions. ( https://www.scribd.com/document/353627133/Breastfeed-Observation-Job-Aid)

 This is why we maintained 2 different names and we advise to use the BFHT for resource-poor, high undernutrition settings where it is useful to have alongside the assessment also some initial advice.

 7. Regarding your advice to proofread the paper to improve the overall writing; we have proofread and now we think this looks fine in this last of the manuscript.
